# Silver diamine fluoride (SDF) used in childhood caries management has potent antifungal activity against oral *Candida* species

**DOI:** 10.1186/s12866-020-01776-w

**Published:** 2020-04-15

**Authors:** Kausar Sadia Fakhruddin, Hiroshi Egusa, Hien Chi Ngo, Chamila Panduwawala, Siripen Pesee, Thenmozhi Venkatachalam, Lakshman Perera Samaranayake

**Affiliations:** 1 Department of Preventive and Restorative Dentistry, University City- Sharjah, University of Sharjah, 27272 United Arab Emirates; 2 Division of Molecular and Regenerative Prosthodontics, Tohoku University Graduate School of Dentistry, 4-1 Seiryo-machi, Aoba-ku, Sendai-city, Miyagi 980-8575 Japan; 3 Faculty of Dentistry, Department of Oral Diagnostic Science, Faculty of Dentistry, Thammasat University, Pathum Thani, Thailand; 4 Sharjah Institute of Medical Research, University City- Sharjah, University of Sharjah, 27272 United Arab Emirates; 5 The University of Hong Kong, Hong Kong, Special Administrative Region China

**Keywords:** *Candida* species, Antifungal efficacy, Silver diamine fluoride, Severe early childhood caries (S ECC), Dentin caries

## Abstract

**Background:**

The microbiome of Severe-Early Childhood Caries (S-ECC), is characterized by an ecosystem comprising bacterial and fungal species, with a predominance of *Candida* species. Hence, an anti-cariogen effective against both bacteria and fungi would be valuable in the management of S-ECC.

Here we evaluate the antifungal effect of silver diamine fluoride (SDF) against 35-clinical yeast isolates (Ten-each of *C. albicans*, *C. krusei, C. tropicalis* and five *C. glabrata* strains) from dentinal caries-lesions from S-ECC.

**Results:**

Disc-diffusion and time-kill assays as well as MIC_50_ and MIC_90_ evaluations against therapeutic concentrations confirmed the broad-spectrum anti-candidal potency of SDF. Ultrastructural images revealed morphologic aberrations of yeast-cell walls on exposure to SDF. All *C. krusei* and *C. glabrata* isolates were significantly more sensitive to SDF, relative to the standard antifungal fluconazole. Further, SDF appears to effectively abrogate filamentation of *C. albicans* even at very low concentrations.

**Conclusions:**

Our data, for the first time, elucidate the antifungal potency of SDF, in addition to its known antibacterial activity, in the management of S-ECC.

## Background

Dental caries, the most prevalent chronic disease of humans, [1] is a highly dynamic pathological process initiated and perpetuated by a polymicrobial community of organisms residing in plaque biofilms [2]. Despite advances in the understanding and the management of the caries process over the last few decades, little headway has been made to eradicate caries, particularly in underserved populations in the developing world [3–5].

The *mutans*-group of streptococci is classically considered as prime movers of the caries process. However, in addition to mutans streptococci several clinical studies have now confirmed the high prevalence of the opportunistic fungal pathogen, *Candida* in plaque-biofilms, particularly in severe early childhood caries (S-ECC) [6–8]. In a recent comprehensive review on S-ECC, Xiao et al. [9] noted the prevalence of *C. albicans* ranging between 60 to 100% in ECC lesions, and others have shown even higher prevalence frequencies in infected deep dentinal lesions [10].

This curious, interspecies, fungal/bacterial cross-kingdom association of acidogenic and aciduric microbes is likely to be due to a sucrose-rich diet of caries prone individuals, that promotes acidification of the plaque biofilm matrix, leading to accelerated caries progression [2, 11, 12]. In a recent review, Pereira et al. (2018) have made a strong case for a significant role of aciduric/acidophilic and acidogenic fungi such as *Candida* in caries progression and deep dentinal caries [13]. Indeed, our own data indicate a very high prevalence of yeast species in over 70% in a Middle East (Emirati) cohort of patients with S-ECC (Fakhruddin et al. unpublished). Therefore, in clinical terms, an effective monotherapy, targeting both the fungal and the bacterial components of the plaque biofilm is urgently needed to manage caries, particularly ECC.

There is a long history of the use of silver compounds in medicine due to its superior antimicrobial properties [14, 15]. Silver is a broad-spectrum chemical with excellent anti-biofilm properties, and hence widely integrated into indwelling devices such as orthopedic prostheses, cardiac devices, and surgical instruments [16–18]. In dentistry too, the susceptibility of several species of oral microbiota to silver-ions has been reported [19, 20], as a result of which it is now incorporated into some dental materials [21]. The exact mechanisms by which particulate silver kills bacteria and fungi are unclear, but it is known that silver causes microbial death by binding to microbial cell wall and membrane components thus causing toxicity and death of the organisms [22].

Relatively recently, a silver-containing compound, silver diamine fluoride -SDF has been added to the anti-microbial armamentarium of dentistry as an attractive therapeutic agent for arresting and preventing dental caries [23]. Silver- ions released from SDF have shown to inhibit cariogenic bacteria, both in the planktonic (suspended) and the biofilm (sessile) phases of growth [24, 25]. For instance, De Almeida and colleagues (2011) used the agar diffusion method to demonstrate the antibacterial effect of SDF against the cariogen, *S. mutans* [26], and a few years later, Targino and his team determined the minimal inhibitory concentration (MIC) and minimum bactericidal concentrations (MBC) of SDF for *S. mutans* [27]. Others too have examined the action of SDF on multispecies bacterial biofilms and reported its high antibacterial activity in such mixed consortia [28–30].

As far as we are aware, there are no studies, to date, in the English language literature assessing the antifungal effect of SDF on *Candida* isolates from plaque biofilm. Although it is well known that SDF is an efficacious antibacterial, its ability to eradicate co-infecting yeasts within the cariogenic biofilm matrix remains unclear. Hence, in this study we evaluated the antifungal effects of SDF against a broad spectrum of common pathogenic *Candida* isolates (*Candida albicans, C. krusei, and C. tropicalis,* and *C. glabrata* isolates) isolate from children with S-ECC.

## Results

### The effect of SDF on different *Candida* species

A total of 35 *Candida* isolates belonging to four different *Candida* spp., viz. *C .albicans* (10 strains)*, C krusei* (10)*, C tropicalis* (10) and *C glabrata* (5) were evaluated for their susceptibility to SDF using the classic, disc diffusion assay. For this purpose filter paper discs impregnated with four different volumes of SDF 2.5 μl, 5 μl, 10 μl, and 15 μl were used and the zones of growth inhibition, in terms of the zone-diameters in millimeters, were measured after 48 h.

**Fig. 1:**
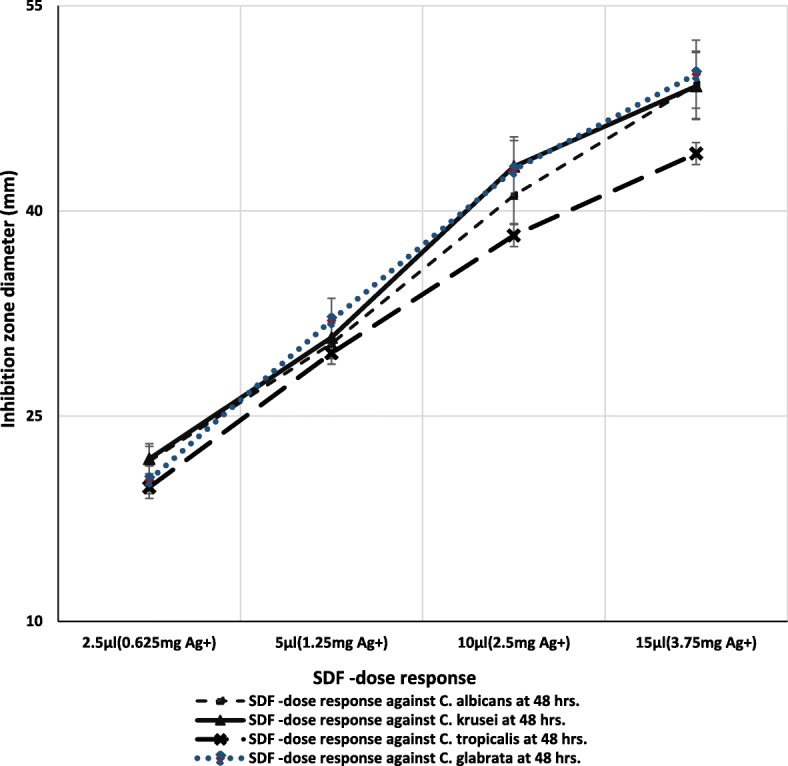
Agar disc diffusion assay: Dose response curves of SDF indicating the zones of growth inhibition (mm) obtained against different concentrations of silver diamine fluoride (SDF; 25% w/v) against four different *Candida* species, *C. albicans* (**a**), *C. krusei* (**b**), *C. tropicalis* (**c**), and *C. glabrata* (**d**); (*p* ≤ 0.001, obtained using ANOVA)

The growth inhibition zone edges due to SDF diffusion were sharply defined and could be readily determined for all the tested strains. We noted increasing concentration of SDF led to increased inhibitory zone diameters in all four species, implying a dose-response effect of SDF on all four tested *Candida* species (*p < 0.001*; Fig. 1). Interspecies variations in the susceptibility of the *Candida* species to SDF was noted with *C. tropicalis*, being relatively more resistant to SDF, according to the diameter of the inhibitory zone of culture growth.

### The relative anti-candidal effect of SDF, fluconazole and amphotericin B

**Fig. 2:**
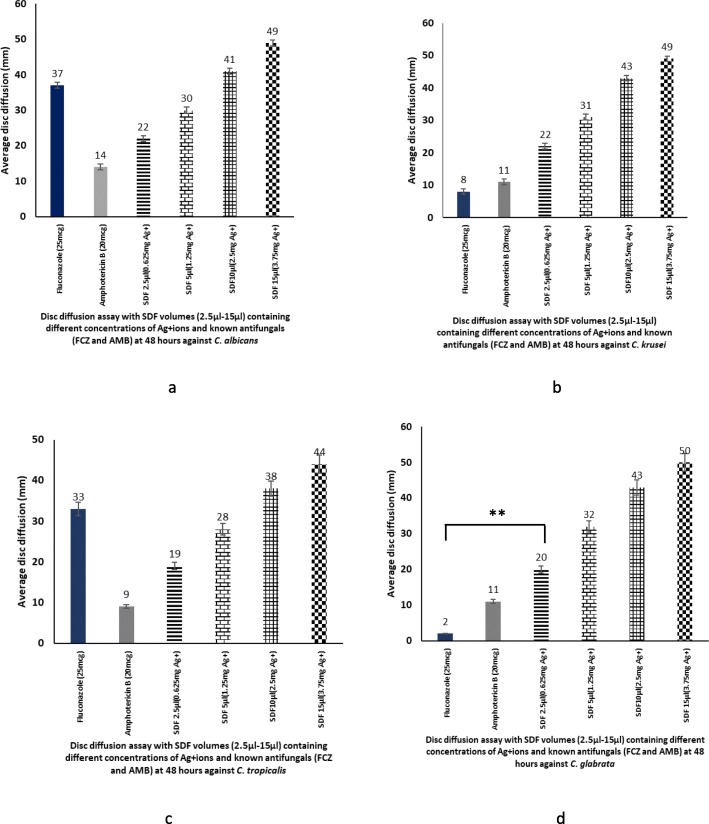
Relative antifungal susceptibility of SDF, four different concentrations of silver ions (0.625 mg, 1.25 mg, 2.5 mg and 3.75 mg Ag^+^), Amphotericin B (20mcg) and Fluconazole (25mcg) against *Candida albicans* (**a**), *C. krusei* (**b**), *C.tropicalis* (**c**) and *C glabrata* (**d**); signficant dfiieences in susceptibility (*p* < 0.001, obatined using ANOVA are asterisked (**)

We also compared the relative antifungal activity of two commonly used antifungals, fluconazole (25 mcg) and amphotericin B (20 mcg) against four different volumes of SDF (2.5 μl, 5 μl, 10 μl, 15 μl) containing viz.: 0.625, 1.25, 2,5, and 3.75 mg of Ag^+^. For broth microdilution assay, the antifungal concentrations of fluconazole and amphotericin B were chosen as per the NCCLS guidelines [31]. In general, the anti-candidal activity of SDF was far superior to the polyene - amphotericin B, and the triaxle - fluconazole (Figs. 2a-d).

On comparing the relative fungicidal activity of fluconazole (25 mcg) and SDF against the ten isolates of *C. tropicalis,* only the highest concentration of SDF proved to be significantly superior to fluconazole, while the other four concentrations of SDF showed lower activity than fluconazole (Fig. 2c). On the other extreme, relative to fluconazole, all ten *C. krusei* isolates and the five *C. glabrata* were significantly more sensitive to all five concentrations of SDF evaluated (*p* ≤ 0.05; Fig. 2b and d).

When the relative antifungal efficacy of amphotericin B vs. SDF was compared, we noted that virtually all four concentrations of SDF significantly inhibited all four *Candida* species we evaluated, compared to a standard 20 mcg concentration of amphotericin B (*p* ≤ 0.05 for all).

### Evaluation of minimum inhibitory concentrations (MIC)

At present, there is neither a documented or universally agreed, standard MIC, nor susceptibility range, nor breakpoint sensitivities for *Candida* species against SDF. In the absence of such information on the relative antifungal efficacy of SDF vs. conventional antifungals (such as fluconazole and amphotericin B) for evaluating MIC, we used the susceptibility range for Amphotericin B and Fluconazole (0.03 to 16 μg/mL and ≤ 8 μg/ml- 16 to 32 μg/ml, respectively) as per the NCCLS recommendations, as the reference point to compare against five different concentrations of SDF. *Candida parapsilosis* ATCC 22019 was used as the control reference strain for this experiment, and its MIC fell within the expected normal range (0.25–2.0 and 0.5–4.0) at 24 h, for Amphotericin B and Fluconazole, respectively [32].

The in vitro antifungal activity of varying volumes of SDF ranging from 0.625 μl–15 μl (equivalent to [Ag+] from 0.156 mg − 3.75 mg, respectively) against four different *Candida* species, are shown in Table 1.

**Table 1:** MIC_50_ and MIC_90_ of Amphotericin B, Fluconazole and SDF against four different *Candida* species (as determined by broth microdilution assay)

*Candida* species (number of isolates)		Silver Diamine Fluoride volumes (μl) (containing mg of silver ions)						SDF μl (mg of silver ions)	Fluconazole (μg)	Amphotericin B (μg)
		**0.625 μl (0.156)**	**1.25** μl **(0.313)**	**2.5 μl (0.625)**	**5 μl (1.25)**	**10 μl (2.5)**	**15 μl (3.75)**	**MIC50 MIC90**	**MIC50 MIC90**	**MIC50 MIC90**
***C.albicans*(10)**	**Number of isolates (percent growth)**	**10 (100%)**	**9 (90%)**	**7 (70%)**	**1 (10%)**	**0 (0%)**	**0 (0%)**	**2.5 (0.625)** **5.0 (1.25)**	**0.25** **1.0**	**0.5** **1.0**
***C.krusei*(10)**		**10 (100%)**	**7 (70%)**	**2 (20%)**	**0 (0%)**	**0 (0%)**	**0 (0%)**	**1.25 (0.313)** **2.5 (0.625)**	**16** **≥32**	**0.25** **0.5**
***C.glabrata*(5)**		**5 (100%)**	**4 (80%)**	**1 (20%)**	**0 (0%)**	**0 (0%)**	**0 (0%)**	**1.25 (0.313)** **2.5 (0.625)**	**8.0** **32**	**0.25** **0.5**
***C.tropicalis (*10)**		**10 (100%)**	**10 (100%)**	**10 (100%)**	**4 (40%)**	**1 (10%)**	**0 (0%)**	**5 (1.25)** **10 (2.5)**	**1.0** **2.0**	**1.0** **2.0**

SDF demonstrated potent fungicidal activity against all tested *Candida* species, including all isolates of *C. krusei* and *C. glabrata* species (known to be resistant to conventional antifungals such as Fluconazole). The MIC_50_ of SDF in the volume range of 1.25 μl − 2.5 μl (equivalent to 0.313 mg–0.625 mg of Ag+, respectively), demonstrated 50% growth inhibition of all *C. albicans, C. krusei, C. glabrata* isolates tested.

However, MIC_50_ of SDF was higher (from 2.5 μl to 5 μl), with the Ag + concentration range between (from 0.625 mg to 1.25 mg) for *C. tropicalis*. The lowest observed MIC of SDF at 1.25 μl volume containing 0.313 mg of Ag + was seen against *C. krusei* and *C. glabrata*. The MIC_90_ of SDF against all the tested *Candida* strains was almost two times higher than the MIC_50_, with the range from 5 μl–10 μl volumes (containing 1.25 mg − 2.5 mg of Ag+, respectively).

**Fig. 3:**
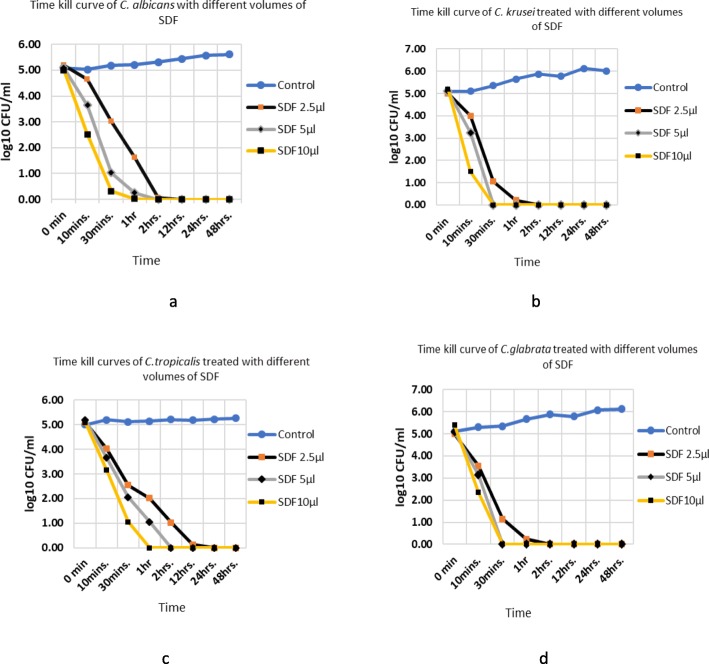
(**a-d**): Time-kill curves of four different volumes of SDF against *C. albicans* (**a**), *C. krusei* (**b**), *C. tropicalis* (**c**) and *C. glabrata* (**d**) (Yeast suspensions of 10^3^ CFU/mL were exposed to different SDF volumes as shown, and log CFU/mL evaluated)

The fungicidal effect of SDF was also determined by time-kill assay at different time-points, over a period of 48-h (Figs. 3a-d). Only representative silver-ions concentrations (0.625–2.5 mg) present in different SDF volumes previously determined by agar diffusion and standard broth microdilution methods were used for these experiments.

Clinical isolates of *C. krusei and C. glabrata* were treated with SDF volume of 5 μl and 10 μl (1.25 mg and 2.5 mg of Ag+) resulted in a complete cell-killing within 30-min. In contrast, *C. tropicalis* strains demonstrated relative resistance to killing, with total fungicidal activity achieved within 60 min of exposure to the identical SDF volumes containing 1.25 mg- 2.5 mg of Ag+. In general, all 35 tested clinical isolates belonging to four different common pathogenic *Candida* species were killed at the lowest volumes of SDF used, containing 2.5 mg Ag+, after 2 h.

### Germ tube assay

**Fig. 4:**
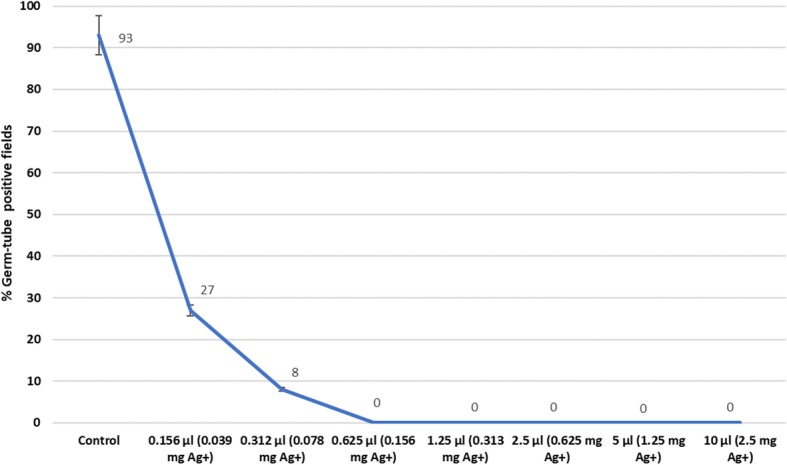
Germ-tube inhibition in *C. albicans* isolates after exposure to SDF (0.156 μl -10 μl volumes; silver ion concentration in parentheses) in fetal bovine serum over a 90 min period; control has no SDF supplement

We evaluated the effect of a range of SDF concentrations on the yeast filamentation using a standard germ tube assay. Two randomly chosen, clinical isolates of *C. albicans* exhibited a profound, dose-response effect when exposed to SDF (Fig. 4). Even at very high dilutions the chemical inhibited yeast germ tube formation, and total abrogation of germ tube formation was seen at 0.039 mg /0.156 μl Ag + concentration.

### Ultrastructural observations

**Fig. 5:**
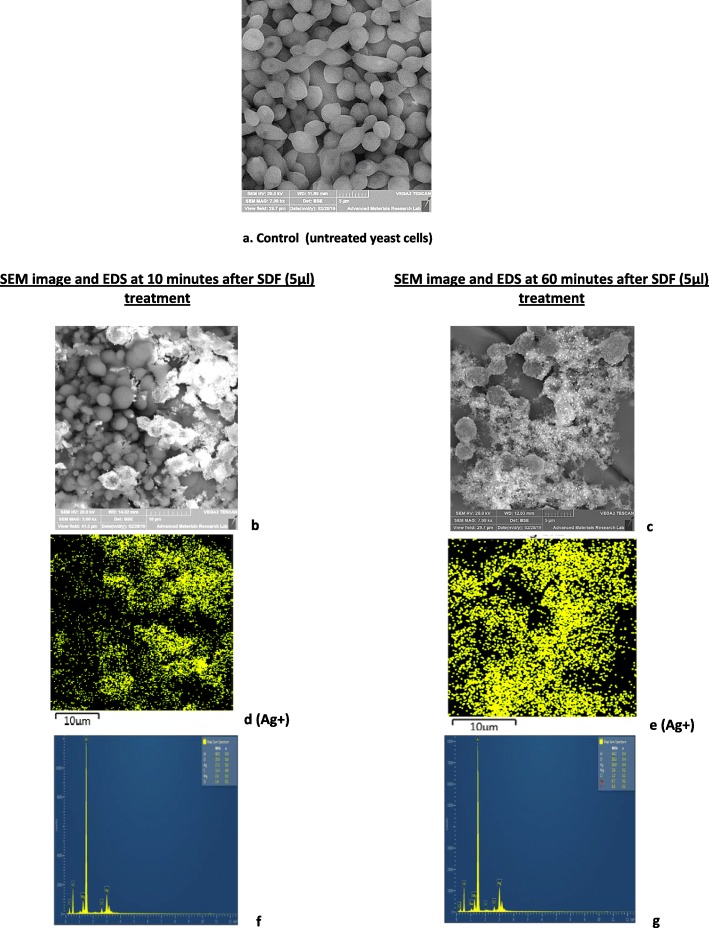
Scanning electron microscopic features and EDS data of a *Candida albicans* strain exposed to SDF(5 μl) obtained after 10 min and 60 min; **a**, Control yeasts unexposed to SDF showing smooth and intact Grecian-vase shaped blastopores. *After 10 min exposure* (**b**, **d**, **f**): Ultrastructural changes of blastopores (**b**) with elemental silver adsorbed/deposited depicted as yellow dots (**d**), and the corresponding EDS profile with Ag + peak (< 1000 counts; *white asterisk*; **f**). *After 60 min exposure*: Amorphous mass of destroyed blastopores (**c**) and intense, and uniform silver adsorption/deposition on blastopores (**e**), and the corresponding EDS profile with a higher Ag + peak are evident (> 1000 counts; white asterisk; **g**)

The antifungal effect of SDF on the cellular, ultrastructural topography of *C. albicans* was examined by scanning electron microscopy. We compared the untreated (control) *C. albicans* and counterparts exposed to SDF (5 μl volume, 1.25 mg Ag + concentration) for 10 min and 1 h (Figs. 5a-g).

The blastopores of *C. albicans* unexposed to SDF generally demonstrated intact, smooth cell walls with Grecian vase morphology and well-defined profiles (Fig. 5a) while structural alterations to the blastopores exposed to SDF were evident after 10 min and the blastopores were virtually amorphous with significant deformation after 60 min exposure to SDF.

Energy Dispersive X-Ray Spectroscopy (EDS) analysis indicated concentrated silver ion deposition on the yeast blastopores after 10 min which became more intense after 60 min (Fig. 5d and e), implying either Ag + ion absorption/ adsorption on the yeast blastopores, that may have contributed to cell deformation and death.

## Discussion

The onset and progression of ECC is rapid and aggressive, leading to the widespread destruction of the enamel as well as the dentinal surfaces of the teeth [3]. Several microbiological studies, including those of ours (unpublished data), have shown that plaque biofilm, particularly from children with ECC, are co-infected with high levels of *mutans*-streptococci, and the common opportunistic oral fungal pathogen *Candida*. It is generally thought that co-habitation of yeasts and bacteria in such an inter-kingdom partnership results in the development of hypervirulent plaque biofilms that accelerates the destruction of the primary dentinal tissues. Hence, any ECC arresting medication must be both anti-bacterial as well as anti-fungal in nature. SDF appears to fulfill this criterion, as our data indicate that apart from its well-known antibacterial action, it is also anti-fungal/candidal in nature. To our knowledge, this is the first study reporting the antifungal potential of SDF against human pathogenic yeasts. Current data demystifies to some extent the pharmacodynamics of SDF, as this antifungal is likely to disrupt cross-kingdom polymicrobial biofilm effectively, by its combinatorial approach. However, further work with polymicrobial bacterial-candidal biofilms is required to confirm this assertion.

The extreme effectiveness of SDF against several human pathogenic species of clinical *Candida* isolates from children with S-ECC can be explained by its ionic silver (Ag+) component. The primary constituents of SDF are ionic silver (Ag+, 25%w/v), fluoride (5%) a re-mineralizing agent and an antibacterial agent, and ammonia (8%), a stabilizing agent [33]. The bioactive form of silver in SDF in its ionized form, as “Ag + ions.” can combine with halides (Cl^−^, F^−^, I^−^), leading to the formation of anionic silver complexes, which are soluble in aqueous media and are highly bio-active. These silver complexes are known to be highly toxic to bacteria [15], as Ag + targets bacteria through a three-pronged approach, by damaging cell-wall structure, denaturing cytoplasmic enzymes, and finally, inhibiting DNA replication [25]. Other workers have noted that the antibacterial effect of SDF is partly due to the interaction of silver-ions with the cell-membranes of cariogenic bacteria, and inactivation of their enzymes, leading to growth inhibition [39, 40]. The ultrastructural features of the SDF-exposed blastopores reported here tend to concur with these observations. Finally, silver ions in SDF is thought to impede dentine-collagen degradation by constraining cathepsins, and thereby arresting caries progression [41, 42].

We also evaluated the relative anti-candidal efficacy of SDF, fluconazole and amphotericin B. Conventionally, the latter two agents are effectively used in the management of oral candidiasis, caused by *C. albicans* and a spectrum of other *Candida* species [43]. Our results suggest that SDF also has an effective spectrum of antifungal activity, even superior to conventional drugs in combating non-albicans *Candida* species such as *Candida krusei* and *Candida glabrata.* Though, our current understanding of the mechanism of action of ionic Ag + on fungal cells is limited, the ultrastructural and EDS observations reported here imply that Ag + avidly binds to the yeast cell walls on exposure to SDF.

In the present study, we characterized the antifungal activity of silver-ions in SDF by an array of methods against four different *Candida* species, i) the agar diffusion assay, ii) microdilution assay, iii) the time-kill assay, iv) germ-tube formation inhibition, and finally, v) ultrastructural analysis by SEM. The disc diffusion method demonstrated clear susceptible zone diameters for all *Candida* species present in clinical isolates, including known fluconazole resistant strains of *C. krusei* and *C. glabrata*. These susceptibility results of Ag + in SDF, intriguingly shows its multi-pronged antifungal potential against a variety of *Candida* strains present in deep niches of the dentine-caries lesions.

Our MIC evaluations indicated the high MIC_50_ and MIC_90_ values for *C. tropicalis* relative to the other three tested species. This could be either due to the growth characteristics (e.g., rapid growth rate) or the target enzymes involved [34, 35]. However, further studies with several *C. tropicalis* strains are required to confirm the observed interspecies variations in SDF sensitivity.

The time-kill curves further confirmed the antifungal potency of SDF. Pharmacokinetics of time-kill observations suggest that the rate of fungicidal activity may be influenced by increased silver-ion concentrations in the suspension. However, the time necessary to achieve a 99.9% reduction in the numbers of CFU/ml observed was higher for *C. tropicalis* compared to other tested species, confirming the MIC observations described above. These data indicate that silver-ions in SDF does display primarily fungicidal effect against a variety of *Candida* species, but possible variations in the target enzyme could account for the interspecies differences in sensitivity. Another possible explanation could be the avidity of the biological ligands of this yeast to SDF [9]. Hall and team (2015), reported that many of the enzymes involved in yeasts-cell wall biogenesis are species-specific and are attractive antifungal targets [36].

*C. albicans* displays dimorphic growth, as the yeast/ blastopore phase and the filamentous or the hyphal phase. The initial step in the filamentation or the hyphal phase is bud formation in the form of germ tubes [46]. The latter attribute of hyphal formation, and its ability to invade soft as well as hard tissue components such as dentinal tubules, is considered an important virulence trait of *C. albicans.* When the impact of varying concentrations of SDF on yeast filamentation was evaluated, we noted that at 0.156 mg of Ag + −(in 0.62 5 μl of SDF), totally abrogated germ tube formation in *C. albicans*. This implies that SDF has the ability to abrogate yeast filamentation even at a very low concentrations, and thereby may hinder penetration of yeasts through dentinal tubules particularly at the advancing front of deep caries lesions.

Finally, our ultrastructural data imply that a clinical formulation of Ag + ions at a concentration of 1.25 mg in 5 μl-SDF volume, robustly binds to the yeast cell walls possibly inactivating and causing cell death. Although we surmised that Ag + were deposited on the blastopore cell walls, it is feasible that fluoride ions may also contribute to the process, in tandem with Ag+. Further studies, are therefore required to clarify this contention.

In clinical terms, it is known that the polymicrobial, inter-kingdom biofilms are covered by a protective layer of extracellular polysaccharides that confers them protection against antimicrobials and chemotherapeutic agents [37, 38]. Therefore, it is crucial to ascertain the activity of SDF within such an ecosystem as our data essentially appertains to the SDF activity against planktonic yeasts. Nevertheless, the above information on the anti-candidal effect of SDF manifestly testifies to the immense potential of SDF as a cheap and effective anti-cariogens.

## Conclusion

Present data derived from 35 caries-associated clinical yeast isolates, for the first time, illustrate the anti-candidal potency of silver diamine fluoride, in addition to its widely known antibacterial activity.

## Methods

The present study was conducted under a protocol approved by the Research Ethics Committee, University of Sharjah (REC-18-02-18-03).

### Yeast isolates

Overall, a total of 85 *Candida* isolates, obtained from 48-yeast positive samples of carious dentin of children with S-ECC were selected for the current study and comprised ten isolates each of *Candida albicans, C. krusei,* and *C. tropicalis,* and five *C. glabrata* isolates (Fakhruddin et al., manuscript in preparation).

The identity of the isolates was reconfirmed by sub-culture on Sabouraud Dextrose Agar (SDA), and the characteristic growth on CHROMagar (HiCrome™ Candida Differential Agar, M1297A) and finally by multiplex PCR (see below).

### Multiplex PCR

The identity of the isolates were further confirmed by Multiplex PCR amplification method, which permitted the identification of six common pathogenic yeast species, namely *C. albicans*, *C. glabrata, C. parapsilosis, C. tropicalis, C. krusei*, and *C. dubliniensis.* The employed method was based on the amplification of two fragments from the ITS1 and ITS2 regions by the combination of two-yeast-specific and six-species-specific primers in a single PCR reaction [39], Table 2. All PCR-reaction products were evaluated by electrophoresis in 2.0% (w/v) agarose gels run at 90 V for 60 mins.

**Table 2:** Amplicon sizes (base pairs) obtained from multiplex PCR amplification using yeast specific (Universal-UNI1 and UNI2) and corresponding species-specific primers for four different *Candida* spp.

Species	Primer	Sequence (5′-3′)	Amplicon size (bp)
	UNI 1 UNI 2	GTCAAACTTGGTCATTTA TTCTTTTCCTCCGCTTATTG	
*C. albicans*	Calb	AGCTGCCGCCAGAGGTCTAA	583/446
*C. tropicalis*	Ctro	GATTTGCTTAATTGCCCCAC	583/507
*C. krusei*	Ckru	CTGGCCGAGCGAACTAGACT	590/169
*C. glabrata*	Cgla	TTGTCTGAGCTCGGAGAGAG	929/839

### Antifungal susceptibility assay by disc diffusion

The antifungal susceptibility of the yeast was evaluated against amphotericin B, fluconazole, and SDF. To prepare the SDF impregnated discs, stocks of sterile filter paper discs stored at 20 °C were allowed to reach room temperature, and then infused with SDF volumes of 2.5 μl, 5 μl, 10 μl, and 15 μl and dried in an oven for an hour at 60 °C before use.

To minimize batch to batch variation, plates were prepared on a single session at ambient temperature, with a constant agar volume of Sabouraud Dextrose Agar (SDA; MH063, Himedia).

All isolates of *Candida albicans*, *C. krusei, C. tropicalis* and *C. glabrata* strains were selected randomly and tested by agar-based diffusion test following CLSI M44-A2 standardized method with some modifications [40]. In brief, a yeast cell suspension of 10^6^ cells/mL from a 24-h old culture was grown in Sabouraud Dextrose broth (ME033, Himedia) and adjusted to 0.5 McFarland standard using a densitometer (Grant Instruments™ Grant Bio™ Densitometer) and spread uniformly using a glass spreader.

The inoculated SDA plate was allowed to dry for 20 min, followed by application, aseptically, of Amphotericin B-20mcg disks (SD233, AP 20mcg, Himedia), Fluconazole-25mcg disks (SD232, FLC 25mcg, Himedia), sterile paper disks with SDF (Thermo Scientific™ Oxoid™ Blank Antimicrobial Susceptibility Discs) and a sterile paper disc (the negative control) using a pair of forceps. The plates were incubated at 37 °C within 15–20 min, after the application of up to a period of 48-h, before evaluation of zones of growth inhibition. Recommended CLSI quality assurance isolates *Candida parapsilosis* ATCC 22019 [25] was tested as a positive control, with each set of experiments.

After 48-h, inhibition zone-diameters were measured to the nearest millimeter at the point where there was a noticeable growth reduction. All experiments were tested in triplicate, on three separate occasions.

### Broth microdilution antifungal susceptibility assay

Minimal inhibitory concentrations (MIC_50_ and MIC_90_) for silver diamine fluoride (SDF) was ascertained following standard methodology set out in CLSI M27-A3 broth microdilution procedure, with some modifications [31, 41] . The modifications include using flat-bottom 96-well microtiter plates (Corning, 3370 Polypropylene Flat Bottom 96 Well) and growth reading determined spectrophotometrically by a microplate reader. As brown discoloration of oxidized silver-ions in SDF makes it challenging to evaluate growth in the wells of the microtiter plates visually.

Amphotericin B (AMB, A2942, Sigma-Aldrich) and Fluconazole (FCZ, ≥98% (HPLC), powder F8929 Sigma-Aldrich) was prepared by dissolving AMB in 5% DMSO and FCZ in sterile distilled water. The solutions were added to RPMI-1640 w/ L-glutamine, 0.2% glucose and 0.165 mol/l MOPS buffer w/o sodium bicarbonate (AT180, RPMI-1640, Himedia) during the time of antifungal exposure to *Candida* spp. Minimum inhibitory effect of AMB and FCZ on clinical isolates were verified over the concentration range of 0.125-64 μg and 0.0312-16 μg, respectively.

Silver Diamine Fluoride complex (Topamine, 25%w/v of silver ions, Product code: DL160.9–1) was obtained from Dentalife Australia Pty. Ltd. Antifungal effects of Ag + particles present in SDF volume-range of 0.156 μl–15 μl were tested over the concentration range between 0.039 mg to 3.75 mg of silver (0.039 mg; 0.078 mg; 0.156 mg; 0.313 mg; 0.625 mg; 1.25 mg; 2.5 mg; 3.75 mg).

For minimum inhibitory concentration (MIC) determinations, all yeast cell suspensions tested were adjusted to a turbidity of 0.5McFarland standard. Subsequently, the cell suspensions were further diluted to a final concentration of 10^3^ cells/ml in the RPMI 1640 medium with 2X serial dilutions of Ag + in SDF, AMB, and FCZ and were pipetted into well of 96-well plates in a standardized manner, and the plates incubated at 37 °C for 24-h. Afterwards, the MICs were determined spectrophotometrically at 490 nm with a microtiter plate reader (BIO-TEK, ELX800, USA). MIC_50_ and MIC_90_ were defined as the lowest drug concentrations that inhibited growth by 50 and 90% compared with drug-free wells, as determined by the absence of turbidity.

In each susceptibility test, QC strain *C. parapsilosis* ATCC 22019 was included. MIC range of 0.5 μg–4 μg of FCZ to *C. parapsilosis* ATCC 22019 was used as a reference.

All tests were replicated on three separate occasions with observations determined independently by two observers.

### Effect of SDF on germ-tube formation

The effect of SDF on the germ-tube formation in *C. albicans* was ascertained following a protocol described by Nair et al., [42] with some modifications. A fresh *C. albicans* inoculum was prepared for the experiment by harvesting a 24-h growth in Sabouraud dextrose agar. A 10^7^ yeast cells/ml suspension was added to fetal bovine serum (F2442, FBS, Sigma-Aldrich) in a microtube. Effects of SDF against germ-tube formation was tested in the volume-range of 0.156 μl–15 μl containing concentration range of silver ions between 0.039 mg to 3.75 mg (0.039 mg; 0.078 mg; 0.156 mg; 0.313 mg; 0.625 mg; 1.25 mg; 2.5 mg; 3.75 mg).

The mixture was then vortexed and incubated aerobically at 37 °C for 90 mins. At 90 min, the formalin solution (HT501640-neutral buffered 10%, Sigma-Aldrich) was added to the mixture to arrest further growth. A drop was removed from each mixture and placed on a glass-slide covered with a coverslip, and germ-tube positive cells were quantified under the microscope (34MP-2 K HD-USB Microscope, under × 40 magnification).

A total of 100 microscopic fields with either germ tube positive or negative blastopores were counted, and the percentage of germ tube positive fields quantified as per the protocol of Nair et al [34]. The germ-tube experiment was repeated twice on different occasions.

### Time-kill assay

Time-kill curves were developed using a protocol described by Klepser et al., with some modifications [43]. The test concentrations of Ag + (0.625 mg; 1.25 mg; 2.5 mg) in SDF volumes of 2.5 μl, 5 μl and 10 μl were assessed at pre-determined time points (0, 30mins, 1 h, 2 h, 12 h and 24 h). A fresh yeast inoculum was prepared for each experiment by harvesting a 24-h growth in Sabouraud dextrose broth, and yeast suspension adjusted, spectrophotometrically, to 0.5McFarland standard (1 × 10^6^ to 5 × 10^6^ CFU/ml).

Yeast suspension (1 ml) was diluted in 9 ml of RPMI 1640 medium with and without SDF, which provides the starting inoculum of 1× 10^5^ to 5 × 10^5^ CFU/ml. After incubation at 37 °C with agitation, a 100 μl aliquot was removed from each solution at predetermined time points and serially diluted (10-folds) in sterile water. From each dilution, a 30 μl aliquot was plated on SDA. Colony counts were subsequently obtained after incubation of the plates at 37 °C, at pre-determined time points. The fungicidal effect was defined as ≥99.9%, or 3-log10-unit, reduction in CFU/ml from the starting inoculum [43, 44]. Time-kill experiments were conducted in duplicate on different occasions.

### Scanning electron microscopy

The effects of the SDF on the ultrastructural features of a single strain of *C. albicans* was investigated using SEM. A randomly selected *C. albicans* strain was incubated in RPMI 1640 at 37 °C for 24- h in a 12-well plate. A yeast suspension of 2.5 × 10^5^ cells in RPMI was prepared into which 5 μl SDF (1.25 mg of silver-ions) was added. The morphological changes of the treated yeast cells were observed after 10- min, and 1-h post-SDF treatment. The antifungal concentration of silver-ions was selected based on the results obtained in the susceptibility testing assays.

Standard methodology with minor modifications was used for SEM analysis of yeast as previously described [45]. Samples of the negative control (untreated cells) and treated cells for 10mins and an hour, respectively, were fixed in 2.5% glutaraldehyde and 4% paraformaldehyde in the presence of cacodylate buffer (pH 6.2) on ice for an hour. Post-fixation of the samples was carried out for 30-min with 1% osmium tetroxide. Samples were gently dehydrated in graded ethanol (30, 50, 70, and 90%). The treated and control samples were then mounted on aluminum stubs and air dried at room temperature, and sputter coated with gold (Polaron SC7640 sputter coater; Thermo VG Scientific, United Kingdom) and observed with an environmental scanning electron microscope (FEI Co., Hillsboro, OR).

Finally, Energy Dispersive X-Ray Spectroscopy (EDS) of the Ag^+^ ion deposition was also carried out for the samples exposed to SDF.

### Statistical analysis

The data obtained from SDF antifungal assays were presented as mean ± standard deviation (SD). MIC_50_, MIC_90,_ and means of inhibition-zone diameters were calculated for each *Candida* strains. The group difference was analyzed using one-way analysis of variance (ANOVA). For all statistical analysis, a *P* value of ≤0.05 was considered statistically significant.

## Data Availability

Data of the present study were analyzed and presented in this published article along with additional files. Additional dataset if required, can be provided by the corresponding author on reasonable request.

## Abbreviations

S-ECC: Severe early childhood caries
SDF: Silver diamine fluoride
MIC: Minimal inhibitory concentrations
AMB: Amphotericin B
FCZ: Fluconazole
SEM: Scanning electron microscopy
PCR: Reverse transcription polymerase chain reaction

## References

[1] Kassebaum NJ, Bernabe E, Dahiya M, Bhandari B, Murray CJ, Marcenes W (2015). Global burden of untreated caries: a systematic review and metaregression. J Dent Res.

[2] Hajishengallis E, Parsaei Y, Klein MI, Koo H (2017). Advances in the microbial etiology and pathogenesis of early childhood caries. Mol Oral Microbiol.

[3] Anil S, Anand PS (2017). Early childhood caries: prevalence, risk factors, and prevention. Front Pediatr.

[4] Caufield PW, Li Y, Bromage TG (2012). Hypoplasia-associated severe early childhood caries--a proposed definition. J Dent Res.

[5] Vargas CM, Ronzio CR (2006). Disparities in early childhood caries. BMC Oral Health.

[6] de Carvalho FG, Silva DS, Hebling J, Spolidorio LC, Spolidorio DMP (2006). Presence of mutans streptococci and Candida spp. in dental plaque/dentine of carious teeth and early childhood caries. Arch Oral Biol.

[7] Yang XQ, Zhang Q, Lu LY, Yang R, Liu Y, Zou J (2012). Genotypic distribution of Candida albicans in dental biofilm of Chinese children associated with severe early childhood caries. Arch Oral Biol.

[8] Raja M, Hannan A, Ali K (2010). Association of oral candidal carriage with dental caries in children. Caries Res.

[9] Xiao J, Huang X, Alkhers N, Alzamil H, Alzoubi S, Wu TT, Castillo DA, Campbell F, Davis J, Herzog K (2018). Candida albicans and early childhood caries: a systematic review and meta-analysis. Caries Res.

[10] Ghasempour M, Sefidgar SAA, Eyzadian H, Gharakhani S (2011). Prevalence of candida albicans in dental plaque and caries lesion of early childhood caries (ECC) according to sampling site. Caspian J Intern Med.

[11] Bowen WH, Burne RA, Wu H, Koo H (2018). Oral biofilms: pathogens, matrix, and Polymicrobial interactions in microenvironments. Trends Microbiol.

[12] Fakhruddin KS, Ngo HC, Samaranayake LP (2019). Cariogenic microbiome and microbiota of the early primary dentition: a contemporary overview. Oral Dis.

[13] Pereira D, Seneviratne CJ, Koga-Ito CY, Samaranayake LP (2018). Is the oral fungal pathogen Candida albicans a cariogen?. Oral Dis.

[14] Klasen HJ (2000). A historical review of the use of silver in the treatment of burns. II. Renewed interest for silver. Burns.

[15] Melaiye A, Youngs WJ (2005). Silver and its application as an antimicrobial agent. Expert Opin Ther Pat.

[16] Morris GV, Kozdryk J, Gregory J, Jeys L (2017). The use of silver-coated orthopaedic implants: are all silvers the same?. Curr Orthop Pract.

[17] Shawcross J, Bakhai A, Ansaripour A, Armstrong J, Lewis D, Agg P, De Godoy R, Blunn G (2017). In vivo biocompatibility and pacing function study of silver ion-based antimicrobial surface technology applied to cardiac pacemakers. Open Heart.

[18] Ammons MC, Ward LS, James GA (2011). Anti-biofilm efficacy of a lactoferrin/xylitol wound hydrogel used in combination with silver wound dressings. Int Wound J.

[19] Youravong N, Carlen A, Teanpaisan R, Dahlén G (2011). Metal-ion susceptibility of oral bacterial species. Lett Appl Microbiol.

[20] Yamamoto K, Ohashi S, Aono M, Kokubo T, Yamada I, Yamauchi J (1996). Antibacterial activity of silver ions implanted in SiO2 filler on oral streptococci. Dent Mater.

[21] Corrêa JM, Mori M, Sanches HL, da Cruz AD, Poiate E, Poiate IAVP (2015). Silver nanoparticles in dental biomaterials. International journal of biomaterials.

[22] Clement JL, Jarrett PS (1994). Antibacterial silver. Met Based Drugs.

[23] Zhao IS, Gao SS, Hiraishi N, Burrow MF, Duangthip D, Mei ML, Lo EC-M, Chu C-H (2018). Mechanisms of silver diamine fluoride on arresting caries: a literature review. Int Dent J.

[24] Chu CH, Mei LEI, Seneviratne CJ, Lo ECM (2012). Effects of silver diamine fluoride on dentine carious lesions induced by Streptococcus mutans and Actinomyces naeslundii biofilms. Int J Paediatr Dent.

[25] Peng JJY, Botelho MG, Matinlinna JP (2012). Silver compounds used in dentistry for caries management: a review. J Dent.

[26] de Almeida LFD, Cavalcanti YW, Valenca AMG (2011). In Vitro Antibacterial Activity Of Silver Diamine Fluoride In Different Concentrations. Acta Odontol Latinoam.

[27] Targino AG, Flores MA, dos Santos Junior VE, de Godoy Bene Bezerra F, de Luna Freire H, Galembeck A, Rosenblatt A (2014). An innovative approach to treating dental decay in children. A new anti-caries agent. J Mater Sci Mater Med.

[28] Lou Y, Darvell BW, Botelho MG (2018). Antibacterial effect of silver Diammine fluoride on cariogenic organisms. J Contemp Dent Pract.

[29] Mei ML, Li Q, Chu C-H, Lo EC-M, Samaranayake LP (2013). Antibacterial effects of silver diamine fluoride on multi-species cariogenic biofilm on caries. Ann Clin Microbiol Antimicrob.

[30] Karched M, Ali D, Ngo H (2019). In vivo antimicrobial activity of silver diammine fluoride on carious lesions in dentin. J Oral Sci.

[31] Pfaller MA, Bale M, Buschelman B, Lancaster M, Espinel-Ingroff A, Rex JH, Rinaldi MG, Cooper CR, McGinnis MR (1995). Quality control guidelines for National Committee for clinical laboratory standards recommended broth macrodilution testing of amphotericin B, fluconazole, and flucytosine. J Clin Microbiol.

[32] Barry AL, Pfaller MA, Brown SD, Espinel-Ingroff A, Ghannoum MA, Knapp C, Rennie RP, Rex JH, Rinaldi MG (2000). Quality control limits for broth microdilution susceptibility tests of ten antifungal agents. J Clin Microbiol.

[33] Horst JA (2018). Silver fluoride as a treatment for dental caries. Adv Dent Res.

[34] Zuza-Alves DL, Silva-Rocha WP, Chaves GM (2017). An update on Candida tropicalis based on basic and clinical approaches. Front Microbiol.

[35] Forastiero A, Mesa-Arango AC, Alastruey-Izquierdo A, Alcazar-Fuoli L, Bernal-Martinez L, Pelaez T, Lopez JF, Grimalt JO, Gomez-Lopez A, Cuesta I (2013). &lt;span class=&quot;named-content genus-species&quot; id=&quot;named-content-1&quot;&gt;*Candida tropicalis*&lt;/span&gt; Antifungal Cross-Resistance Is Related to Different Azole Target (Erg11p) Modifications. Antimicrob Agents Chemother.

[36] Hall RA (2015). Dressed to impress: impact of environmental adaptation on the Candida albicans cell wall. Mol Microbiol.

[37] Berger D, Rakhamimova A, Pollack A, Loewy Z. Oral Biofilms: Development, Control, and Analysis. High Throughput. 2018;**7**(3):1–8. 10.3390/ht7030024 PMC6163956 30200379

[38] Ramage G, Rajendran R, Sherry L, Williams C (2012). Fungal biofilm resistance. Int J Microbiol.

[39] Carvalho A, Costa-De-Oliveira S, Martins ML, Pina-Vaz C, Rodrigues AG, Ludovico P, Rodrigues F (2007). Multiplex PCR identification of eight clinically relevant Candida species. Med Mycol.

[40] Arendrup MC, Park S, Brown S, Pfaller M, Perlin DS (2011). Evaluation of CLSI M44-A2 disk diffusion and associated breakpoint testing of caspofungin and micafungin using a well-characterized panel of wild-type and fks hot spot mutant Candida isolates. Antimicrob Agents Chemother.

[41] Pfaller MA, Espinel-Ingroff A, Boyken L, Hollis RJ, Kroeger J, Messer SA, Tendolkar S, Diekema DJ (2011). Comparison of the broth microdilution (BMD) method of the European committee on antimicrobial susceptibility testing with the 24-hour CLSI BMD method for testing susceptibility of Candida species to fluconazole, posaconazole, and voriconazole by use of epidemiological cutoff values. J Clin Microbiol.

[42] NAIR RG, ANIL S, SAMARANAYAKE LP (2001). The effect of oral bacteria on Candida albicans germ-tube formationNote. APMIS.

[43] Klepser ME, Ernst EJ, Lewis RE, Ernst ME, Pfaller MA (1998). Influence of test conditions on antifungal time-kill curve results: proposal for standardized methods. Antimicrob Agents Chemother.

[44] Pfaller MA, Sheehan DJ, Rex JH (2004). Determination of fungicidal activities against yeasts and molds: lessons learned from bactericidal testing and the need for standardization. Clin Microbiol Rev.

[45] Samaranayake LP, MacFarlane TW (1980). An in-vitro study of the adherence of Candida albicans to acrylic surfaces. Arch Oral Biol.

[46] Samaranayake LP (2018). Essential microbiology for dentistry, 5th edn. Elsevier: Philadelphia, 9 pp. 255–266.

